# Access to electronic health knowledge in five countries in Africa: a descriptive study

**DOI:** 10.1186/1472-6963-7-72

**Published:** 2007-05-17

**Authors:** Helen Smith, Hasifa Bukirwa, Oscar Mukasa, Paul Snell, Sylvester Adeh-Nsoh, Selemani Mbuyita, Masanja Honorati, Bright Orji, Paul Garner

**Affiliations:** 1 International Health Group, Liverpool School of Tropical Medicine, UK; 2 Malaria Research Group, Makarere University, Uganda; 3 Ifakara Health Research & Development Centre, Tanzania; 4 Medical Research Council Laboratories, Banjul, The Gambia; 5 Holy Trinity Development Foundation, Holy Trinity Foundation Hospital, Cameroon; 6 Department of Health Promotion and Education, College of Medicine, University of Ibadan, Nigeria

## Abstract

**Background:**

Access to medical literature in developing countries is helped by open access publishing and initiatives to allow free access to subscription only journals. The effectiveness of these initiatives in Africa has not been assessed. This study describes awareness, reported use and factors influencing use of on-line medical literature via free access initiatives.

**Methods:**

Descriptive study in four teaching hospitals in Cameroon, Nigeria, Tanzania and Uganda plus one externally funded research institution in The Gambia. Survey with postgraduate doctors and research scientists to determine Internet access patterns, reported awareness of on-line medical information and free access initiatives; semi structured interviews with a sub-sample of survey participants to explore factors influencing use.

**Results:**

In the four African teaching hospitals, 70% of the 305 postgraduate doctors reported textbooks as their main source of information; 66% had used the Internet for health information in the last week. In two hospitals, Internet cafés were the main Internet access point. For researchers at the externally-funded research institution, electronic resources were their main source, and almost all had used the Internet in the last week. Across all 333 respondents, 90% had heard of PubMed, 78% of BMJ on line, 49% the Cochrane Library, 47% HINARI, and 19% BioMedCentral. HINARI use correlates with accessing the Internet on computers located in institutions. Qualitative data suggested there are difficulties logging into HINARI and that sometimes it is librarians that limit access to passwords.

**Conclusion:**

Text books remain an important resource for postgraduate doctors in training. Internet use is common, but awareness of free-access initiatives is limited. HINARI and other initiatives could be more effective with strong institutional endorsement and management to promote and ensure access.

## Background

Cheaper hardware and increasing Internet coverage in sub-Saharan Africa potentially increases access to reliable, up to date medical literature, but prohibitive commercial on-line subscription costs for journals are a major constraint. In 2002 the World Health Organization (WHO) launched the HINARI programme, which allows free access to full text journals in academic and medical institutions, teaching hospitals, and government offices in low income countries provided they register with the scheme [1,2]. This benefits many countries, although India, China and South Africa, for example, are excluded as they do not meet the definition of low income. Alternatives to HINARI include initiatives where access is negotiated at country level, and paid for by national government or donor funds, such as the Programme for the Enhancement of Research Information (PERI) [3]. Open access journals eliminate this problem, and a few publishers offer priced journals for free or at reduced price.

As we could find virtually no empirical data about access in Africa [4,5], we supervised a small survey by email of 55 health professionals in 36 developing countries and asked respondents to retrieve the full text (pdf) of selected journal articles [6]. 45% of respondents were in countries eligible for free access to priced journals via HINARI. 80% of all respondents could download a pdf article freely available on the BMJ website, but fewer were able to download pdfs from priced journals including a Lancet article (42%) and a Cochrane review (16%). Respondents stated that failure to retrieve was because they did not have a subscription to the journal. This prompted us to explore awareness of HINARI and whether users were experiencing technical problems in using it. The study objective was to measure awareness, reported use and factors influencing use of on-line medical information and free access initiatives in postgraduate doctors in training in Africa.

## Methods

### Study sites and participants

We first established a research network, through existing collaborative links and with advice from the World Health Organization, from East and West Africa. In each country, the researcher purposefully selected four large government funded teaching hospitals with postgraduate programmes. The participants were postgraduate doctors with a basic medical degree, studying for a higher qualification. We chose this group as they represent the medical opinion leaders of the future. We included researchers at an externally funded medical research institution located in The Gambia, where access to medical literature was likely to be optimal, as a comparator (see Table 1).

**Table 1:** Study sites, participants, and HINARI uptake^1^

Category	Country	Study site	Registered Post Grad. doctors^2^	Number approached for survey	Number completed survey	Internet facilities available	HINARI log ins per month	HINARI pdf downloads per month
National postgraduate institutions	Cameroon	Faculty of Medicine University of Yaoundé 1	146	89^2^	60	Library	<1	<1
	Nigeria	College of Medicine, University of Lagos	206	156	108^3^	Library	100	219
	Tanzania	Muhimbili University College of Health Sciences	48	48	46	Library; postgraduate computer lab	91	189
	Uganda	Mulago National Referral & Teaching Hospital	130	110^4^	91	Library; postgraduate & faculty computer rooms	350	607
Externally funded research institution	The Gambia	Medical Research Council Laboratories	70^5^	60	28	All office computers connected	221	562

	Total				333			

^1 ^Average number of log ins and downloads from May to December 2005 (data provided by WHO); ^2 ^57 out of the country in training; ^3 ^48 of the selected postgraduate students were out of the campus for professional examinations;^4 ^20 away from the hospital; ^5 ^target population was research scientists

### Data collection methods and sampling

We used a self-administered questionnaire, drawing on similar examples [7,8]. We asked about Internet use for medical literature, and whether they had heard of specific online services (additional file 1). We identified options from a pilot study in Uganda: PubMed/Medline, HINARI, BMJ website, the Cochrane Library, Medscape and BioMedCentral. We piloted questionnaires in each country with postgraduate doctors not included in the actual study sample. We approached the relevant teaching hospital administration to obtain an up to date list and contact details of all registered postgraduate doctors, then drew up a list of those available to participate in the survey (Table 1). We traced these individuals and physically handed the questionnaire to them. In the four national postgraduate institutions respondents were followed up and questionnaires collected directly from them; in Lagos, those living outside campus returned completed questionnaires to the Chief Resident doctor's office. At the MRC laboratories email reminders were sent to all participants and completed questionnaires were collected by hand, or delivered by hand or internal mail; two were returned electronically.

We conducted semi-structured interviews with a sub-sample of survey participants who indicated they used online resources frequently (three at each site), and other key informants (one librarian and one senior opinion leader at each site), to explore in more detail the factors influencing use of Internet-based medical journals (additional file 2). No survey respondents were interviewed in The Gambia. Interviews were conducted by trained social scientists in each country. Notes were taken during the interview and read back to respondents at the end of the interview for clarification or modification.

### Data analysis

Survey data were checked for completeness on site, and hardcopies sent to Tanzania for double entry into a single database using DMSys and the analysis done centrally using a predefined analytical plan. We used frequency tables to describe data across countries, and where appropriate explored relationships between categorical variables to provide insight to the observed results. We explored the influence of year of qualification, speed of Internet connection, and usual place of Internet access on 'ever use' of initiatives using Chi-square tests, but found no significant association. The whole research team discussed site reports and overall analysis during an analysis workshop.

Two trained social scientists (HS, SM) carried out a thematic analysis of the qualitative data using methods of Framework approach [9] and MAXqda software [10] to manage data coding, searching and retrieval (additional file 3). Key informant interviews conducted in French (in Cameroon) were translated into English in-country. An initial coding frame was developed in Liverpool and applied independently by both researchers. After a simple content analysis, the dimensions of frequently used codes were explored and documented in matrices. Both researchers categorised data, discussed emerging themes and came to a consensus in the analysis workshop.

### Ethical approval

Ethical approval was obtained in all participating countries (Cameroon: Faculty; Uganda: National Council for Science and Technology; Tanzania: National Institute for Medical Research; Nigeria: Research and Ethics Committee, Lagos University Teaching Hospital; The Gambia: Ethics Committee of The Gambia Government and the MRC) and also from Liverpool School of Tropical Medicine in the UK.

## Results

### Survey

Three hundred and thirty three of the 463 people completed the questionnaire (72%). Of the respondents 25% were women (n = 85; data missing n = 13); the distribution was similar between countries, with Uganda as the lowest, with 15% women respondents. In the four national postgraduate institutions the majority (66%) of postgraduate doctors had been qualified for between 5 and 10 years (data missing n = 13); mostly in medicine (n = 117) or surgery (n = 97). At the externally funded research institution most staff were research scientists.

In the national postgraduate institutions, 70% of respondents reported textbooks were their main source of health information (table 2); contrasting with the externally funded research institution, where 85% of research scientists reported E-resources as their main supply.

**Table 2:** Main source of health and medical information*

		Textbooks (%)	E-resources**	Hard copy journals
National postgraduate institution	Yaoundé University	37 (62)	22 (37)	1 (2)
	Lagos College of Medicine	81 (76)	23 (22)	2 (2)
	Muhimbili College	32 (70)	14 (30)	0 (0)
	Mulago Teaching Hospital	63 (70)	27 (30)	0 (0)
	Sub-total	213 (70)	86 (29)	3 (1)
Externally funded research institution	MRC Laboratories	2 (7)	23 (85)	2 (7)

* Missing data n = 4

** Electronic journals and general internet resources

In the national postgraduate institutions, the majority of respondents (63%) had accessed the Internet for health or medical literature in the last week (table 3). Access was less frequent in Lagos (Nigeria), where a quarter of respondents had not used the Internet for medical information for more than a month.

**Table 3:** Last time used the internet to access health or medical literature*

		Within a week (%)	Within a month	More than a month ago
National postgraduate institutions	Yaoundé University	36 (61)	17 (29)	6 (10)
	Lagos College of Medicine	43 (41)	36 (34)	27 (25)
	Muhimbili College	41 (89)	5 (11)	0 (0)
	Mulago Teaching Hospital	68 (77)	15 (17)	5 (6)
	Subtotal	188 (63)	73 (24)	38 (13)
Externally funded research institution	MRC Laboratories	27 (96)	1 (4)	0 (0)

* Missing data n = 6

Access to the Internet varied between sites. At the MRC Laboratories, most respondents had their own Internet connection in their office (89%), but this was rare in the national postgraduate institutions (table 4). In Mulago (Uganda) and Muhimbili (Tanzania) access in communal areas predominated; whereas in Lagos (Nigeria) and Yaoundé (Cameroon) the majority of respondents used Internet cafés.

**Table 4:** Usual place of internet use*

		Own office (%)	Shared/communal place	Own home	Internet café
National Postgraduate institutions	Yaoundé University	1 (2)	4 (7)	1 (2)	54 (90)
	Lagos College of Medicine	1 (2)	13 (12)	25 (24)	67 (63)
	Muhimbili College	5 (11)	27 (59)	10 (22)	4 (9)
	Mulago Teaching Hospital	2 (2)	69 (78)	2 (2)	15 (17)
	Subtotal	9 (3)	113 (38)	38 (13)	140 (47)
Externally funded research institution	MRC Laboratories	25 (89)	2 (7)	1 (4)	0 (0)

* Missing data n = 5

More than half of the respondents (59%) reported to obtain their last full text journal article by downloading directly from the Internet. The use of CD-ROMs to obtain full-text articles appears to be very low (table 5).

**Table 5:** Method of retrieval of full text article on most recent occasion*

		Direct from Internet (%)	Photocopy from journal in library	CD ROM	Request copy from library
National postgraduate institution	Yaoundé University	33 (55)	15 (25)	8 (13)	4 (7)
	Lagos College of Medicine	55 (53)	35 (34)	11 (11)	3 (3)
	Muhimbili College	29 (63)	11 (24)	0 (0)	6 (13)
	Mulago Teaching Hospital	51 (58)	10 (11)	5 (6)	22 (25)
	Subtotal	168 (56)	71 (24)	24 (8)	35 (12)
Externally funded research institution	MRC Laboratories	24 (86)	1 (3)	0 (0)	3 (11)

* Missing data n = 7

Awareness of online health information initiatives varied across sites. In the national institutions, most respondents had heard of PubMed (90%) and the BMJ website (78%), but were less likely to have heard of the Cochrane Library, HINARI or BioMedCentral. Compared to other sites fewer postgraduate doctors in Yaoundé (Cameroon) had heard of the BMJ (42%). Awareness of the Cochrane Library was highest in Mulago (Uganda), and more postgraduate doctors in Muhimbili (Tanzania) and Mulago (Uganda) had heard of HINARI compared to other sites. At the MRC Laboratories, most researchers had heard of HINARI, PubMed and BMJ, but were unfamiliar with Medscape (table 6).

**Table 6:** Awareness of on line initiatives: respondents who had "ever head of" each initiative

		PubMed/Medline (%)	BMJ‡	Cochrane Library	Medscape	HINARI	BMC†
National postgrad. institutes	Yaoundé University	55 (92)	25 (42)	13 (22)	28 (47)	18 (30)	9 (15)
	Lagos College of Medicine	87 (82)	94 (79)	50 (47)	48 (45)	26 (24)	13 (12)
	Muhimbili College	43 (93)	42 (91)	26 (56)	32 (70)	33 (72)	19 (41)
	Mulago Teaching Hospital	85 (97)	73 (83)	69 (79)	53 (61)	64 (73)	17 (19)
	Sub-total	270/300 (90)	234/300 (78)	146/299 (49)	161/299 (54)	141/300 (47)	58/299 (19)
Externally funded research institute	MRC Laboratories	27 (100)	22 (79)	15 (56)	11 (41)	28 (100)	18 (64)

†BioMedCentral open access journal; ‡British Medical Journal website

### Qualitative study

Three categories of data were identified in the analysis, relating to: a) the responsibilities of publishers or providers of online health information; b) the organisation of access at institutions; and c) individual experiences of online initiatives. Across these categories we identified three main themes, described below.

#### 1. The 'free full text' myth

Respondents from each study site complained they could rarely access full text articles online. Medline appeared to be the most popular way of obtaining journal articles, but users frequently described not being able to obtain full text articles, a typical response was, "I use PubMed...but very often I am not satisfied as mostly abstracts are presented and full text articles are not free". Respondents frequently described locating an article of interest "only to find that it needs a subscription". They reported problems accessing articles for free even within so-called 'free initiatives', for example, "HINARI has a common password for this institution, but users are discouraged because they say at times some cost must be incurred if full text is requested". Others described circumventing high subscription fees by using their contacts in overseas institutions to obtain articles from priced journals, including the Cochrane Library.

Respondents also described their frustration at publisher delay in providing recent articles for free, a typical comment was that publishers "claim free access after a period of time, yet often this proves not to be the case". Suggestions included publishers should "tighten up their policies" on free access, and that publishers should "improve their service so that they [articles] are available when they say they are".

#### 2. Problems with passwords and controlled sites

Postgraduate doctors described difficulties logging in to websites that require a password, including specific problems logging into HINARI. Some stated that "passwords do not guarantee opening a website". Because of this, many expressed a preference for websites that do not require log in to access journals, for example: "...PubMed is heavily used, its simply because its free...they don't require a password...for other databases like HINARI, you have to have a username and password so its not accessible...". Several postgraduate doctors thought that access would be made easier if passwords could be used outside of institutional boundaries.

Respondents were concerned about the management of passwords to access electronic journals within their institutions, explaining that it was difficult to obtain passwords from administration or librarians, who either do not make them readily available, or who "may not always be available to provide them". Others referred to a tension between library staff (as gate-keepers of passwords) and Internet users, for example, a librarian explained "...use of institutional password is not convenient for users...because they need the librarians to access the password...those who heard from their colleagues about HINARI but do not meet us when they come to the library usually get offended". Another librarian expressed concern that users "appear to be in a hurry" and don't consult the librarians before accessing websites; and this was perceived as users "boycotting" the librarians. There were several requests from postgraduate doctors for passwords to be made more available via the university website, published regularly in college newsletters, or displayed on library notice boards rather than being "hidden" from users.

#### 3. Improving access at institutions

Almost all respondents mentioned problems with hardware, Internet connections and computing facilities at their institutions. Many comments related to interrupted power supply, typically, "one of the things that disturbs the connection is the electricity; if the electricity goes off, then we don't have a connection" and "power offs present another obstacle to accessing Internet based information effectively". Others commented on the quality of the connection, typical responses were "at times the connection is really slow and even slower for sites with images" and "there are also cases where logging in to some databases like HINARI takes long, after waiting for so long you get a message that the connection has failed". Others described a need for institutions to improve computing facilities including the number of computers available for users and reducing the cost of machines for postgraduates.

Many respondents commented that poor connections and power supply interruptions also had implications for downloading articles: "pdf format is preferable to HTML in places where connectivity is good" and "it is better for one to at least access full text if connectivity is slow and this is only possible for HTML format, but if connectivity is fine then one can go for pdf format". Postgraduate doctors also described problems printing and saving articles, particularly in PubMed: "I have difficulties printing and saving while using PubMed, I need to create an MS Word environment to be able to print".

Opinion leaders and postgraduate doctors thought it was the responsibility of their institution to "provide information about the websites it is subscribed to" and raise awareness about the free online resources available to them. A need for formal orientation to available online resources and accessing initiative websites was also expressed by respondents across the study sites.

## Discussion

This is a small study and dependent on the respondents answering accurately. We were careful to try and establish the denominator at each hospital, and had a reasonable response rate. The study sites are among the largest government funded training institutions in East and West Africa; Internet access is more likely to be available at these institutions. The results of the survey may be generalisable to other large teaching hospitals in countries in Africa with similar facilities.

We were surprised that postgraduate doctors in training were so dependent on text books as their main source of information. However, this probably reflects the structure of most postgraduate medical courses around clinical competence and knowledge, with limited expectations on understanding of current research and policy debates. Postgraduate training for doctors in the study countries involves a research based dissertation, but accessing up to date online articles might not be a strict requirement. Nevertheless, Internet access was high, and particularly in Muhimbili and Mulago hospitals, which have a more extensive research portfolio.

The high use of commercial outlets was astonishing, with Internet cafés being the main place of Internet use for postgraduate doctors in Lagos teaching hospital and Yaoundé University. This may well reflect the limited investment in cabling and other infrastructure in these postgraduate teaching institutions; other institutions may have invested more. The question specifically stated this was last place of use "for medical and scientific literature" so we are fairly sure this is how they responded, but were unable to validate this against actual access. The majority of all respondents stated that the last time they retrieved a full text article was through the Internet, illustrating the importance of this source of medical knowledge for postgraduates in these countries.

The results show low use of CD-ROM resources across all sites and this could be because this type of resource is complicated to distribute, can become out of date rapidly, and there are fewer organisations supplying material in this format now.

We found awareness of initiatives varied widely by country and initiative. Low awareness of the BMJ website in Yaoundé University, Cameroon could be to do with provision of material in English, although we do not have specific data on this. Awareness of the Cochrane Library appeared higher in Mulago hospital, Uganda compared to other sites; this could be attributed to awareness raising workshops on evidence informed practice by visiting researchers.

The log in data from HINARI (table 1) correlate with higher reported awareness of HINARI and use of a computer within an institution. Fewer people in Lagos and Yaoundé had heard of HINARI, and used Internet cafés for access-presumably as institutional access was poor. The most use made of HINARI was through the externally funded research institution in The Gambia and Mulago hospital, Uganda where HINARI has been extensively promoted via institutional training.

The qualitative findings indicate that provision of passwords could be better managed and teaching hospitals could do more to publicise the various electronic resources available to students and staff. What was surprising was the degree to which librarians controlled access: it seemed that some librarians saw it as their job to provide the articles for the doctors, rather than allow the doctors to access websites themselves and in these institutions passwords were not freely available. The qualitative data also suggest users have difficulty with passwords and access, and that HINARI may wish to review the ease of use of its interface. Respondents described problems accessing free full text articles via Medline which may be because they are not logged into HINARI. Once logged in, users can search for articles through Medline in the usual way and have direct access to the full text. Staff at the WHO are currently improving the interface and publicising HINARI to help improve access to passwords in institutions.

Our findings suggest power interruptions and inadequate computing facilities continue to be major constraints; combined with apparent problems logging in to Internet based initiatives and difficulties acquiring passwords from institutional gate keepers, these factors represent considerable barriers to the effective use of initiatives providing access to online journals at the teaching hospitals in our study. Relatively small investments in connectivity which are carefully managed by institutions will increase access to large amounts of information. In addition, HINARI would be more effective with strong institutional endorsement and management to promote and ensure access.

## Conclusion

In postgraduate doctors in four selected national postgraduate institutions in Africa:

• High and regular use of the Internet is reported, and Internet cafés are the most important Internet access point for two of the four institutions studied

• Awareness of free access initiatives is variable; it is highest for PubMed and lowest for BioMedCentral

• HINARI helps access in some research led institutions, but there are problems with organising distribution of passwords in others, and some users report difficulties making HINARI work.

• A high level of connectivity and use can be organised in countries in Africa, as demonstrated by the data from an externally funded research institution located in one country.

## Competing interests

The author(s) declare that they have no competing interests.

## Authors' contributions

HS identified the study question and developed the protocol with HB and PG. OM, SM, SA, MH, BO collected, analysed and interpreted country data. HS, HB and PG conducted initial cross-country data analysis and all authors interpreted this data. HS and PG drafted the paper and all authors contributed to the final manuscript. HS is the guarantor.

## Pre-publication history

The pre-publication history for this paper can be accessed here:



## Supplementary Material

Additional File 1 Questionnaire. The self-administered questionnaire used in this study. Click here for file

Additional File 2 Topic guides. Topic guides for the semi-structured interviews conducted in this study. Click here for file

Additional File 3 Thematic analysis. A detailed description of the thematic analysis of qualitative data. Click here for file
