# Supplementary material for: Access to electronic health knowledge in five countries in Africa: a descriptive study
Source: BMC Health Serv Res. 2007 May 17;7:72. doi: 10.1186/1472-6963-7-72 (PMC1885254; doi:10.1186/1472-6963-7-72)
Supplement: Additional File 1 — Questionnaire. The self-administered questionnaire used in this study. [file 1472-6963-7-72-S1.pdf]

Additional file 1 - Questionnaire

1. Gender

Male		Female	

2. Year qualified as a medical doctor

3. Completed years in postgraduate training — Years

4. What was your main responsibility in your previous job? (Tick ONE only)

No previous job		Public health/management		Research	
Clinical care		Teaching			

5. What are you training for?

Surgery and ENT		Medicine and related		Paediatrics	
O and G		Diagnostics/lab sciences		Public health/epidemiology	

6. Currently, what is your main source of health and medical information? (Tick ONE only)

Textbooks		Electronic journals and CDs		Hardcopy journals	
Colleagues		Drug company information		General internet resources	

7. Have you ever used the Internet?

Yes		No	

7b. If No, can you mention any particular reason(s)

➔➔ If you have NEVER used the Internet, please end the survey now. Thank you for taking time to complete the above questions.

8. How often do you use the Internet to access health or medical literature?

Several times a week	☐	Few times a month	☐	Few times a year	☐

9. When was the last time you used the Internet to access health or medical literature?

Within a week	☐	Within a month	☐	Longer than a month ago	☐

10. Where do you generally access the Internet? (Tick ONE only)

Own office	☐	Shared access/communal place	☐	Library	☐
Own home	☐	At friend or relative	☐	Internet cafe	☐

11. What is the Internet connection like?

Good – rapid webpage access	☐	Bit slow – but I got what I wanted	☐
Unsatisfactory - kept disconnecting	☐		

12. Where did you access the Internet last time? (Tick ONE only)

Own office	☐	Shared access/communal place	☐	Library	☐
Own home	☐	At friend or relative	☐	Internet cafe	☐

13. Have you ever attended formal training on:

13a. How to use a computer?	Yes	☐	No	☐
13b. Searching the Internet?	Yes	☐	No	☐
13c. Using online health and medical resources?	Yes	☐	No	☐

14. How would you rate your skills in the following tasks?

14a. How to use a computer	Basic ☐	Intermediate ☐	Advanced ☐
14b. Internet or database search skills	Basic ☐	Intermediate ☐	Advanced ☐

15. The last time you needed a specific full text article from a medical or health journal, which of these best describes the way you obtained it? (Tick ONE only)

Acquire directly online from the Internet ☐	Find a journal in the library and photocopy article ☐
Obtain from CD ROM ☐	Request a copy from the library or information officer ☐

➔ There are currently various online initiatives providing free or reduced cost access to full text journal articles; the following questions relate to these.

16. The Health InterNetwork Access to Research Initiative (HINARI) provides free full text access to over 2500 international medical and health journals online via an institutional password.

16a. Have you ever heard about HINARI? Yes ☐ No ☐

16b. Do you use HINARI to access online medical journals and resources?

No ☐	Tried and failed to access ☐	Several times a week ☐
Few times a month ☐	Few times a year ☐	

16c. Please give any comments or experiences concerning HINARI:

17. **BioMedCentral** (BMC) is an online initiative that provides free full text access to its electronic medical journals via “open access publishing”, where authors of papers, not the readers, bear the cost.

17a. Have you ever heard about BioMedCentral?

Yes		No	

17b. Do you use BioMedCentral online medical journals?

No		Tried and failed to access		Several times a week	
Few times a month		Few times a year			

17c. Please give any comments or experiences concerning BioMedCentral:

18. The publisher of the **British Medical Journal** (BMJ) provides free full text access to its journals directly via its website.

18a. Have you ever heard about BMJ online?

Yes		No	

18b. Do you use BMJ online to access full text articles?

No		Tried and failed to access		Several times a week	
Few times a month		Few times a year			

18c. Please give any comments or experiences concerning BMJ online:

19. **Medscape** is an information gateway that provides access to medical literature via free individualised registration and password.

19a. Have you ever heard about Medscape?

Yes	☐	No	☐

19b. Do you use Medscape to access online medical journals and resources?

No	☐	Tried and failed to access	☐	Several times a week	☐
Few times a month	☐	Few times a year	☐		

19c. Please give any comments or experiences concerning Medscape:

20. The **Cochrane Library** consists of a regularly updated collection of evidence-based medicine databases including a database of systematic reviews.

20a. Have you ever heard about the Cochrane Library?

Yes	☐	No	☐

20b. Do you use the Cochrane Library?

No	☐	Tried and failed to access	☐	Several times a week	☐
Few times a month	☐	Few times a year	☐		

20c. Which version of the Cochrane Library do you use most?

Internet	☐	CD ROM	☐

20d. Please give any comments or experiences concerning the Cochrane Library:

21. **Medline or PubMed** is a database containing abstracts of articles and citations to biomedical journals published worldwide.

21a. Have you ever heard about Medline or PubMed?

Yes		No	

21b. Do you use Medline or PubMed?

No		Tried and failed to access		Several times a week	
Few times a month		Few times a year			

21c. Which version of Medline or PubMed do you use most?

Internet		CD ROM	

21d. Please give any comments or experiences concerning Medline or PubMed:

22. Are there any other online health information sources you use regularly? (**Please state below**)

THANK YOU FOR YOUR TIME
