# Supplementary material for: Access to electronic health knowledge in five countries in Africa: a descriptive study
Source: BMC Health Serv Res. 2007 May 17;7:72. doi: 10.1186/1472-6963-7-72 (PMC1885254; doi:10.1186/1472-6963-7-72)
Supplement: Additional File 2 — Topic guides. Topic guides for the semi-structured interviews conducted in this study. [file 1472-6963-7-72-S2.pdf]

# Additional file 2 - Topic guides

### Interview with librarian

1. As you are aware, there are many ways to access medical journal articles via the Internet – can you tell me how these initiatives are being used at this library?

   *Prompt - for each initiative mentioned, ask about student access to the initiative, the quality of connection, passwords, which initiatives used most and why?*

2. What could be done locally to improve access to these resources?

   *Prompt if not mentioned:*
   *Technical aspects (connection, webpage accessibility, password access)*
   *Human aspects (computer or search skills)*
   *Organisational aspects (password availability, access to computers)*

3. What could be done by online journal providers to improve their services?

   *Prompt if not mentioned:*
   *Technical aspects (connection, webpage accessibility, password access)*
   *Human aspects (content, interface, format of downloads)*
   *Organisational aspects (password availability, IP address recognition)*

**Remember to use probing questions to encourage a respondent to give more information; some examples of probing questions:**

**‘Can you tell me a little more about that?’**

**‘Why do you think that?’**

**‘How do you feel about that?’**

**‘How does that work?’**

**‘I’m not sure I know what you mean’**

**‘Why do you think that is?’**

**Interview with senior opinion leader**

1. As you are aware, there are many ways to access medical journal articles via the Internet – can you tell me about your institution's experience?

   *Prompt - for each initiative mentioned, ask about the quality of connection, passwords, usability of the website*

2. What could be done locally to improve your institution's access to these resources?

   *Prompt if not mentioned:*
   *Technical aspects (connection, webpage accessibility, password access)*
   *Human aspects (computer or search skills)*
   *Organisational aspects (password availability, access to computers)*

3. What could be done by online journal providers to improve their services?

   *Prompt if not mentioned:*
   *Technical aspects (connection, webpage accessibility, password access)*
   *Human aspects (content, interface, format of downloads)*
   *Organisational aspects (password availability, IP address recognition)*

**Remember to use probing questions to encourage a respondent to give more information; some examples of probing questions:**

**'Can you tell me a little more about that?'**

**'Why do you think that?'**

**'How do you feel about that?'**

**'How does that work?'**

**'I'm not sure I know what you mean'**

**'Why do you think that is?'**

**Interview with postgraduate medical students from survey sample**

1. You have indicated that you access medical journal articles via the Internet – can you tell me about your experience?

   *Prompt - for each initiative mentioned, ask about where they access computers and the internet, how access is organised, the quality of connection, passwords, usability of the website, which initiatives do they prefer to use, why?*

2. What could be done locally to improve your access to these resources?

   *Prompt if not mentioned:*
   *Technical aspects (connection, webpage accessibility, password access)*
   *Human aspects (computer or search skills)*
   *Organisational aspects (password availability, access to computers)*

3. What could be done by online journal providers to improve their services?

   *Prompt if not mentioned:*
   *Technical aspects (connection, webpage accessibility, password access)*
   *Human aspects (content, interface, format of downloads)*
   *Organisational aspects (password availability, IP address recognition)*

**Remember to use probing questions to encourage a respondent to give more information; some examples of probing questions:**

**‘Can you tell me a little more about that?’**

**‘Why do you think that?’**

**‘How do you feel about that?’**

**‘How does that work?’**

**‘I’m not sure I know what you mean’**

**‘Why do you think that is?’**
