# Supplementary material for: Access to electronic health knowledge in five countries in Africa: a descriptive study
Source: BMC Health Serv Res. 2007 May 17;7:72. doi: 10.1186/1472-6963-7-72 (PMC1885254; doi:10.1186/1472-6963-7-72)
Supplement: Additional File 3 — Thematic analysis. A detailed description of the thematic analysis of qualitative data. [file 1472-6963-7-72-S3.pdf]

# Additional file 3 - Thematic analysis

We developed an initial coding index comprising 29 codes (based on recurrent issues in interview transcripts, the research aims, and topic guides).

Both analysts independently applied this coding index to the data using MAXqda[1]software: transcripts were imported; a code system was set up; and codes applied to data electronically. We compared coding and it was fairly consistent between analysts; disagreements were resolved by discussion.

We then used functions available in MAXqda to search and browse coded sections of text. The code matrix browser helped identify frequently used codes across all interviews, and the code relation browser allowed us to determine where codes overlapped. From this we were able to collapse some codes, re-name others and then re-group them into three broad categories (Table 1). Categorisation involved several rounds of discussion between the two analysts.

Table 1. Broad categories of data

| Broad categories | | | |
|---|---|---|---|
| | a) publisher or provider responsibilities | b) organisation of access at institutions | c) individual experiences of online initiatives |
| Codes | Access to abstracts only | Computer-user ratio | Registration/Subscription procedure |
| | Registration/Subscription procedure | Electricity supply | Access to abstracts only |
| | Journal availability | Internet connections | Users discouraged by charges for full text articles |
| | Suggestions to improve online services | Equipment & computing facilities | Preferences for websites with no passwords |
| | Providers should create interactive training | Awareness raising /publicity of available initiatives | Logging on outside institutions |
| | Webpage & article format | Access via librarian | Social browsing |
| | Inconvenience of institutional passwords | Password availability | |
| | Cost of accessing full text articles is high | Logging on outside institutions | |
| | Speed of access | Institutions should organise formal training for users | |

[1] MAXqda version 2; 2005.

We developed a matrix (in MS Word[2]) for each category which included summaries of coded text to allow us to look across all coded data; table 2 shows a matrix extract for the category ‘publisher or provider responsibilities’:

Table 2. Extract from a matrix

| a) publisher or provider responsibilities (category) | | |
|---|---|---|
| Code | Summary of coded text | Interview ID |
| Access to abstracts | Pubmed/Medline is free and easy to use, but you can only access abstracts | Int 3_Nigeria_OL |
| | Accesses BMJ for full text articles, but frustrated with PubMed as has never accessed a full articles, only abstracts. | Int 21_Tanzania_Dr |
| | Uses Medline but not satisfied as full text articles are not free. | Int 15_Cameroon_Dr |
| | PubMed easy to use but problem is it only contains abstracts | Int 11_Uganda_Dr |
| | Accesses PubMed but mostly gets abstracts, wishes for full text. | Int 6_Nigeria_Dr |
| Registration & subscription | Many problems subscribing to HINARI, lengthy process | Int 4_Nigeria_Lib |
| | HINARI should have similar log on to BMJ; friendlier steps; reduces problem of username and institutional password | Int 3_Nigeria_OL |
| | Difficulty accessing HINARI, got password from friend, webpage wouldn’t open, tried several times. | Int 6_Nigeria_Dr |
| | Passwords inconvenient, doesn’t guarantee opening a site, logging in to HINARI takes long time | Int 11_Uganda_Dr |
| | So many sites require passwords and this becomes confusing | Int 21_Tanzania_Dr |

Matrices enabled us to look across the data and identify patterns or connections and interpret the whole dataset. Both analysts compared and contrasted possible patterns in the data, and identified emerging themes. Consensus was reached on final themes by discussion and by returning to the original objectives of the research. The three main themes identified were: 1) the free full text myth; 2) problems with passwords and controlled sites; and 3) improving access at institutions. Table 3 shows illustrative quotes from the theme ‘the free full text myth’.

[2] We are aware that the latest version of MAXqda (2007) supports the production of tables, matrices, maps and other graphical representation of data.

Table 3. Illustrative quotes from the theme 'the free full text myth':

| Theme 'the free full text myth' |
|---|
| Extracts of coded data |
| "…of course I agree that there are some of the websites which are free, but some of the information or most of it is available in the protected websites and you can't get full text because you are not a subscriber.." (Int_22) |
| "HINARI has a common password for this institution. But users are discouraged because they say at times some cost must be incurred if full text is requested" (Int_13) |
| "There are others like British Medical Journal; I access full articles with no subscription. These are the sites I'm mostly using. There are the sites like PUBMED, actually I don't like it completely, because I have never accessed full article, it's just abstracts. So because of this, I don't want to subscribe." (Int_21) |
| "I equally use Medline and Pubmed but very often I am not satisfied as mostly abstracts are presented and full text articles are not free." (Int_15) |
| "PubMed is easy to use as long as one has the basic skills to search but the problem is that they contain only abstract." (Int_11) |
| "I also have access to Pubmed but it gives mainly abstracts most times with few full text articles. I wish I could get full text articles…" (Int_6) |
| "...Another aspect is that we get information but when you want to access some of the information's you find that they are protected. So to some extent the Internet are free but not all what you want is free; some of the information is protected so we can't have access to it." (Int_22) |
| "Yes, I do find HINARI very easy to use - if the article is available for free. It is always disappointing when you get to the particular article you need only to find that it needs a subscription… I do wish that HINARI would only show those journals that are fully free - it can be so frustrating." (Int_1) |
| "Another problem is that you locate an article and discover some articles are not free (not all of the articles accessed on HINARI are free)." (Int_4) |
| "HINARI should only include journals that do offer truly free access. This also applies to the journals that offer free access after a period of time. Some publishers claim free access after this period of time, yet often this proves not to be the case. Publishers should tighten up on their policies and make them clearer - either change the time delay to be more accurate, or improve their services so that they are available when they say they are" (Int_1) |
| "Also, I use Lancet. But you need to subscribe at a fee or buy the journal. The subscription fee is not affordable. My colleague abroad helps to subscribe for me… As regards Cochran Library, I have access to CD-ROM 2004 edition…okay, I access it through a friend who is a reviewer so I do not bother to access it through the net." (Int_5) |
| "I know that most staff and Resident Doctors cannot afford the subscription fees. But some of our staff with international contacts… those who schooled abroad most times access the initiative through their previous institutions or professional associations." (Int_4) |
